# Intergenerational Coparenting Across Physical Distance: Grandparenting Styles, Parental Remote Parenting, and Mental Health Among Left-Behind Children in China

**DOI:** 10.3390/bs16071234

**Published:** 2026-07-20

**Authors:** Yean Wang, Wenjie Li, Yifan Du, Yixin Wu

**Affiliations:** School of Government, Beijing Normal University, Beijing 100875, China; echowang@bnu.edu.cn (Y.W.); luoluo0903@mail.bnu.edu.cn (W.L.); ddup0425@163.com (Y.D.)

**Keywords:** left-behind children, mental health, grandparenting style, parental remote parenting, intergenerational coparenting

## Abstract

The family care of left-behind children often combines in-person grandparental caregiving with parental remote parenting; yet, how these two forms of care jointly relate to children’s mental health remains underexplored. Drawing on family systems theory, this study used data from 6317 rural left-behind children cared for by grandparents in the 2023 Hubei Province Survey of Vulnerable Children. Latent class analysis (LCA) and regression models were applied to identify grandparenting styles, parental remote parenting, and their interaction. Grandparenting styles were identified as warmly controlling, indifferent punitive, and mildly interventional. Compared with the mildly interventional style, the warmly controlling style was associated with higher resilience and lower psychological distress, whereas the indifferent punitive style showed the opposite pattern. Parental remote parenting was associated with higher resilience and lower psychological distress, but its strength varied across grandparenting contexts. In the indifferent punitive style, its positive association with resilience was weaker; in the warmly controlling style, its negative association with psychological distress was weaker. These findings suggest that parental remote parenting serves as a conditional family resource embedded in grandparental care environments, contributing to the mental health of left-behind children. The study shows the asymmetric role of intergenerational coparenting in psychological resilience and distress.

## 1. Introduction

Ongoing urbanization and parental labor migration have significantly transformed the parenting structures within rural Chinese families. Prolonged parent–child separation due to labor migration has made grandparents the primary caregivers for many rural left-behind children. At the same time, the development of mobile communications and digital media has enabled parents to re-engage in their children’s lives via telephone, video calls, and instant messaging, thereby facilitating parental remote parenting. Consequently, the upbringing of left-behind children is no longer solely contingent on parental physical presence but has evolved into an intergenerational coparenting mechanism, where grandparenting styles coexist with parental remote parenting (Lin et al., 2025; C. Zhou et al., 2021).

Although studies have separately explored the role of grandparenting styles and parental remote parenting in child development, findings remain inconsistent. Reviews and meta-analyses of grandparenting indicate significant heterogeneity in its impact on children’s mental health; supportive grandparenting styles are typically associated with lower levels of depression and anxiety, whereas more negative grandparenting styles are linked to greater internalizing problems (Y. Wang et al., 2024; Zhao et al., 2025). Research on parental remote parenting generally finds that higher-quality or more frequent parent–child communication is associated with greater psychological resilience and fewer behavioral problems (F. Lu et al., 2025; Xie et al., 2024; C. Zhou et al., 2021). Together, these studies suggest both factors influence the mental health of left-behind children, but their interaction remains unclear. Grandparenting is often treated as a single continuous variable, with few studies categorizing it by combinations of supportive, controlling, and negative behaviors. Moreover, research tends to focus on negative outcomes like depression and anxiety, neglecting positive adaptive traits such as resilience. Parental remote parenting is usually viewed as an independent predictor rather than within the family context of grandparenting. Thus, current research has yet to clarify how parental remote parenting relates to children’s mental health across different grandparenting styles.

Family systems theory offers a useful framework for understanding this issue, viewing families as interconnected systems rather than isolated individuals. It identifies three interacting subsystems in families with left-behind children: grandparent–child, parent–child, and parent–grandparent. These ongoing interactions shape the child’s developmental environment, providing a basis to examine how grandparenting styles and parental remote parenting jointly affect mental health outcomes.

This study draws on data from the 2023 Hubei Province Survey of Vulnerable Children, encompassing 6317 left-behind children mainly cared for by grandparents. Latent class analysis identifies distinct grandparenting styles, while regression models assess their links to psychological resilience and distress, considering the moderating effect of parental remote parenting. Models control for grade level to address developmental differences across the 3–18 age range. This study integrates grandparenting and parental remote parenting within a family systems framework, capturing their nested interplay. Using latent class analysis, we reveal heterogeneity in grandparenting styles. By examining both psychological resilience and distress as dimensions of mental health, the study moves beyond a deficit-only perspective to highlight asymmetric mechanisms of intergenerational coparenting, providing actionable insights for targeted mental health interventions.

## 2. Literature Review and Theoretical Framework

### 2.1. The Mental Health of Left-Behind Children

Children’s mental health has become a significant global public health concern (Lawrance, 2024). About one in seven children and adolescents aged 10 to 19 experience mental health problems, the most common of which are anxiety, depression, and behavioral disorders (World Health Organization, 2024). These conditions not only hinder children’s normal growth and development but also have lasting negative effects on their professional capabilities, social adaptation, and quality of life in adulthood (Xiao et al., 2023). Therefore, early identification and intervention in children’s mental health is a critical global issue that requires urgent attention.

Mental health issues of left-behind children have long drawn public concern. In China, the world’s largest developing country, this rural subgroup is especially significant in children’s mental health research (Qi & Wu, 2024). A 2021 survey by the Institute of Psychology, Chinese Academy of Sciences, found depression rates of 28.5% and excessive anxiety at 27.7% among left-behind children, both higher than in non-left-behind peers, highlighting systemic risks to rural development (Fang et al., 2023). Existing research indicates that, compared to non-left-behind children, left-behind children exhibit higher depression, anxiety, and lower self-esteem, as well as a reduced sense of well-being (R. Chen & Zhou, 2021), which significantly impacts their social, academic, and emotional functioning (Lin et al., 2025).

The core cause of mental health issues among left-behind children lies in fundamental changes in family parenting structure (Zhao et al., 2025). Prolonged separation from parents, who are children’s primary emotional attachments, may indirectly lead to insufficient emotional support and a disruption of parent–child attachment and communication, which severely undermines the fundamental safeguards for children’s mental health development (Y. Lu et al., 2021; Yeung et al., 2001), thereby affecting the development of their emotional regulation and social adaptation skills (Pilkauskas & Dunifon, 2016). Although grandparents provide daily care (Renny & Reddy, 2016), traditional attitudes often prioritize nurturing over education, worsening children’s internalized psychological problems (Pilkauskas & Dunifon, 2016). Thus, optimizing grandparenting styles and enhancing parental remote parenting are key to supporting left-behind children’s mental health.

The mental health issues of rural left-behind children have evolved from individual or family concerns into a significant structural problem impacting China’s coordinated urban-rural development and social equity (Q. Zhou et al., 2026). This study focuses on children’s mental health, addressing China’s social development needs and offering insights for tackling children’s mental health challenges linked to population migration worldwide.

### 2.2. Grandparenting Styles and Children’s Mental Health

Grandparenting styles refer to the sustained involvement of grandparents as primary caregivers in children’s daily living contexts (R. Chen et al., 2025). In families with left-behind children, the emotional interactions between grandparents and children constitute the children’s proximal environment, exerting a continuous influence on the development of the children’s mental health (Spence et al., 2001). This research defines children’s mental health as a two-dimensional construct: firstly, psychological resilience, as a form of positive adaptation, reflecting children’s ability to recover and grow in stressful situations (Hu & Gan, 2008); secondly, psychological distress, as a negative symptom, encompasses levels of depression, anxiety, and stress (Lovibond & Lovibond, 1995). This two-dimensional construct transcends traditional, problem-oriented single indicators, enabling a more comprehensive understanding of children’s mental health development.

In the context of prolonged parental absence, grandparenting style is essentially a complex blend of multidimensional behaviors rather than a single trait. Existing research has attempted to classify grandparenting styles across different dimensions. From the perspective of responsibility allocation (Ju & Zhang, 2022), whereas such classifications emphasize the intergenerational transfer of parenting responsibilities, they, to some extent, overlook the dynamic nature of interaction. From the perspective of intervention causes (Pruchno & Johnson, 1996), whereas the classification reveals the contextual background of grandparenting style formation, it fails to reveal its operational mechanisms within the family system. From the dimension of parenting styles (Cho & Garcia, 2025), whereas the classification captures key behavioral characteristics to some extent, it treats behavioral elements in isolation, making it difficult to reflect their integrated patterns in real-life contexts. To address the above issues, this study employs a person-centered latent class analysis. By examining the combinatorial patterns of multidimensional behaviors, it identifies different types of grandparenting styles, providing a more accurate reflection of the actual parenting environment in which children find themselves.

Current research generally finds that the relationship between grandparenting styles and children’s mental health is not unidirectional but rather exhibits distinct divergent characteristics. On the one hand, grandparents can provide children with sustained emotional support through consistent companionship and frequent interaction, promoting the development of secure attachment, emotional regulation skills, and psychological resilience (Arafat et al., 2022; Liang & Van Leeuwen, 2025). On the other hand, in certain contexts, grandparenting styles may also be significantly associated with children’s anxiety, depression, and behavioral problems (R. Chen et al., 2025; Yang & Liu, 2020). Based on this, the present study proposes the following hypothesis:

**H1.**  *Grandparenting styles are differentially associated with left-behind children’s mental health, as indicated by psychological resilience and distress.*

### 2.3. Parental Remote Parenting and Children’s Mental Health

Existing research on left-behind children’s mental health has primarily relied on analytical frameworks such as attachment theory, ecosystem theory, and family systems theory. Attachment theory emphasizes that parent–child communication is beneficial to the physical and mental development of left-behind children, providing new insights into its key role in their developmental process (Liu et al., 2025). However, its excessive focus on the relationship between parents and children makes it difficult to explain the complex dynamics of multiple attachment relationships within a grandparenting context. Although ecosystem theory emphasizes the impact of multidimensional environments—such as parents, interpersonal relationships, and the individual—on children’s mental health development (Sun et al., 2026), it inadequately explains the interactive mechanisms between family subsystems, limiting its ability to account for intergenerational coparenting in rural Chinese families. A common limitation of these theories is their treatment of families of left-behind children as static, singular environments, ignoring the family’s complexity as a dynamic, interactive system.

Family Systems Theory offers a suitable framework for analyzing children’s mental health and family interactions. It emphasizes that families are composed of interdependent subsystems, functioning as a whole greater than the sum of its parts, with individual development shaped by interactions among these subsystems (Bowen, 1992; Minuchin, 1985). In the context of Chinese families with left-behind children, this theory explains interactions among three key subsystems. First, the grandparent-child subsystem, the child’s most direct nurturing environment, provides daily emotional support, behavioral norms, and value guidance. Second, the parent-child subsystem maintains a parental psychological presence through remote media, offering emotional and behavioral guidance. Third, the parent-grandparent subsystem highlights how coordination or conflict between parents and grandparents jointly influences children’s mental health (F. Lu et al., 2026; Wu et al., 2024). Emotional attunement and dyadic emotion regulation in parent–child interactions are fundamental to children’s socioemotional development (Christou & Flora, 2025). Although physical separation may disrupt these processes, remote communication could partially sustain emotional connectedness if parents remain emotionally responsive and engaged during virtual contact. Thus, parental remote parenting, like in-person parenting, relies on relational and emotional mechanisms shaped by the broader family caregiving context.

Prior research on remote parenting, transnational parenting, and migration-related family processes has conceptualized this construct through multiple dimensions, such as communication quality, emotional responsiveness, parental monitoring, emotional support, educational guidance, and involvement in family decision-making (Jia & Tian, 2010; Mazzucato, 2015). Drawing on theories such as distant mothering and mobile phone parenting (Wu et al., 2024), this study defines parental remote parenting as how often parents interact with their children via phone, video, or other media while physically absent. This frequency-based measure was chosen because it was the most reliably captured in a large-scale census and, from a family systems perspective, reflects the continuity and strength of the parent–child bond despite separation.

Research shows a systematic link between the frequency of parental remote parenting and children’s mental health. Frequent parental remote parenting is associated with greater psychological resilience (L. Wang & Mesman, 2015), lower depression and anxiety (Y. Jiang, 2025), and better adaptive abilities and social behavior (Liang & Van Leeuwen, 2025; L. Wang & Mesman, 2015). This protective effect may stem from maintaining parents’ psychological presence and partially compensating for emotional gaps caused by physical absence, allowing children to receive ongoing emotional support and guidance. Additionally, frequent remote parenting could enhance family social capital, enriching children’s social support networks (M. Jiang et al., 2026). Thus, parental remote parenting plays a role in emotional support and relationship maintenance, providing left-behind children with psychological protection and resources for positive adaptation.

From a family systems perspective, parental remote parenting does not function independently, but its effectiveness is highly dependent on the child’s family parenting environment (Bowen, 1992; Minuchin, 1985). Its effects may vary based on grandparenting style. On the one hand, when grandparenting style has a positive impact on the child, parental remote parenting may create a synergistic effect with in-person care, jointly enhancing the child’s psychological resilience (Liang & Van Leeuwen, 2025). On the other hand, when grandparenting style has a negative impact on children, parental remote parenting may play a compensatory role by providing emotional comfort and behavioral guidance. However, frequent punitive interactions may hinder the effectiveness of remote parenting as a psychological resource, potentially weakening its impact (Zheng & Gao, 2021). 

Based on Family Systems Theory, this study regards parental remote parenting as a conditional resource whose effectiveness may depend on the grandparent-child subsystem’s characteristics, proposing the following core hypothesis:

**H2.**  *Parental remote parenting moderates the relationship between grandparenting styles and the mental health of left-behind children.*

**Figure 1:**
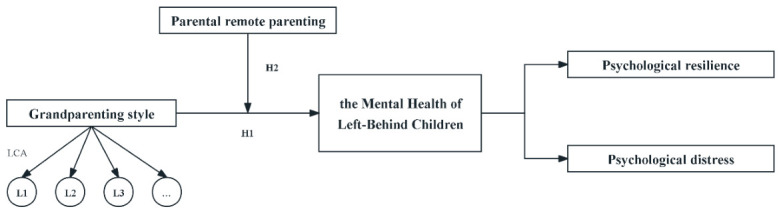
Conceptual model. Note: “…” indicates latent classes to be identified from data, not pre-specified categories.

In summary, the mental health of left-behind children is jointly influenced by grandparenting styles and parental remote parenting. This research views grandparenting style as a multidimensional behavioral pattern with potential heterogeneity and explores its relationship with children’s psychological resilience and distress. Parental remote parenting is introduced as a key contextual factor to analyze its moderating effect on different grandparenting styles. Accordingly, the study proposes a conceptual model (see Figure 1), illustrating the interactions among grandparenting styles, parental remote parenting, and the mental health of left-behind children.

## 3. Materials and Methods

### 3.1. Data Source and Sample

Hubei Province, located in central China, experiences common rural labor migration across regions. In many left-behind families, grandparents or other relatives provide daily childcare, making skipped-generation caregiving prevalent. This setting is ideal for studying the socioeconomic and migration backgrounds of left-behind children’s families (Song et al., 2018). This study was part of a large-scale census survey on the mental health of children in difficult circumstances in Hubei, conducted from 7 December to 18 December 2023, with support from the Hubei Provincial Department of Civil Affairs. The survey was implemented with the help of Child Directors, grassroots officials responsible for child and women’s affairs in villages and communities. A total of 28,699 questionnaires were distributed, covering left-behind children, migrant children, children with serious illnesses or disabilities, orphans, and other children in difficult circumstances.

The research protocol was approved by the Research Ethics Committee of Central China Normal University and supported by the Children’s Welfare Division of the Hubei Provincial Department of Civil Affairs. Data were collected via questionnaires overseen by local child welfare authorities in village and community settings. Children able to comprehend and complete the questionnaire independently were encouraged to do so without assistance. Younger participants or those encountering difficulties with reading or understanding the questions received support from caregivers, parents, or local child welfare officials, tailored to their specific contextual needs. This assistance included reading questions aloud, explaining response options, or helping complete the forms. In instances where children’s capacity for independent response was limited, data were obtained from caregivers, parents, or local officials.

This analysis concentrated on left-behind children meeting the following inclusion criteria: both parents had migrated for employment for a cumulative duration exceeding six months; the primary caregiver was a paternal or maternal grandparent; the child’s age ranged from 3 to 18 years; and complete data were available for key variables. The final sample included 6317 children across various educational stages: preschool (*N* = 541), lower primary school (grades 1–3; *N* = 1580), upper primary school (grades 4–6; *N* = 2390), junior high school (*N* = 1572), and senior secondary, vocational secondary, or technical school (*N* = 234). Thus, the dataset covers children and adolescents across diverse developmental stages.

### 3.2. Measurement

The independent variable, grandparenting styles, was measured using the Chinese version of the Family Upbringing Questionnaire developed by Carlo Perris, translated by Yue et al. (1993). Items, rated on a four-point scale, assessed supportive, controlling, and negative behaviors. Since these behaviors often co-occur in families, no total score was calculated. Instead, the 14 items served as indicators in a latent class analysis to identify grandparenting style types. Specific items are reported in Appendix Table A1.

The dependent variable was children’s mental health, measured by psychological resilience and distress. Resilience was assessed using a 17-item scale rated on a six-point scale (see Appendix Table A2), with the mean score representing overall resilience. Higher scores indicated greater resilience. The scale showed high internal consistency in this sample, with a Cronbach’s α of 0.985. Exploratory factor analysis further indicated good structural consistency with a KMO value of 0.980 and a significant Bartlett’s test. Principal factor extraction yielded one factor with an eigenvalue of 13.776, explaining 81.03% of the variance. Factor loadings ranged from 0.754 to 0.933. Psychological distress was measured by the DASS-21 (see Appendix Table A3), which covers symptoms of depression, anxiety, and stress (Cronbach’s α = 0.974). Items were rated on a four-point scale, with the mean score serving as the composite score. Higher scores indicated greater distress.

The moderating variable, frequency of parental remote parenting (see Appendix Table A4), was measured by the average of two items evaluating contact frequency between the child and each parent. This variable reflects the continuity and strength of the parent–child relationship despite physical separation. After reverse coding, higher scores indicated more frequent remote contact. Given the sample’s wide age range, age and grade were included as covariates in the regression models to control for developmental differences. Control variables comprised gender, household registration, parental marital status, and parental education. County fixed effects were also incorporated.

### 3.3. Analytical Strategy

Latent class analysis (LCA) was conducted in R 4.5.2 to identify latent types of grandparenting styles. Grandparenting includes multiple dimensions, such as supportive, controlling, and negative behaviors. Families with similar total scores may show different behavioral configurations. As a person-centered method, LCA identifies latent classes based on individuals’ joint response patterns across multiple items, making it suitable for examining the heterogeneous structure of grandparenting styles. Models with one to six classes were fitted using the 14 grandparenting items. The final number of classes was determined by comparing the log-likelihood, AIC, BIC, adjusted BIC, entropy, and class proportions. After identifying latent classes, regression models were used to examine the associations between grandparenting styles and parental remote parenting with the mental health of left-behind children. The models further tested the interaction between grandparenting styles and parental remote parenting to assess whether the association between parental remote parenting and mental health varied across grandparenting contexts.

## 4. Results

### 4.1. Descriptive Statistics

The sample of 6317 left-behind children (mean age 10.38) showed above-average psychological resilience (4.637), low distress (1.24), and above-average parental remote parenting (4.85). The demographic characteristics of the sample are shown in Table 1. The gender distribution was largely balanced, with the majority of students in the upper primary and junior high schools; the predominant household registration type was agricultural, and the majority of parents were married. The educational attainment of both fathers and mothers was predominantly at the lower secondary level, accounting for 69.95% and 72.06%, respectively.

### 4.2. Latent Class Analysis of Grandparenting Styles

**Figure 2:**
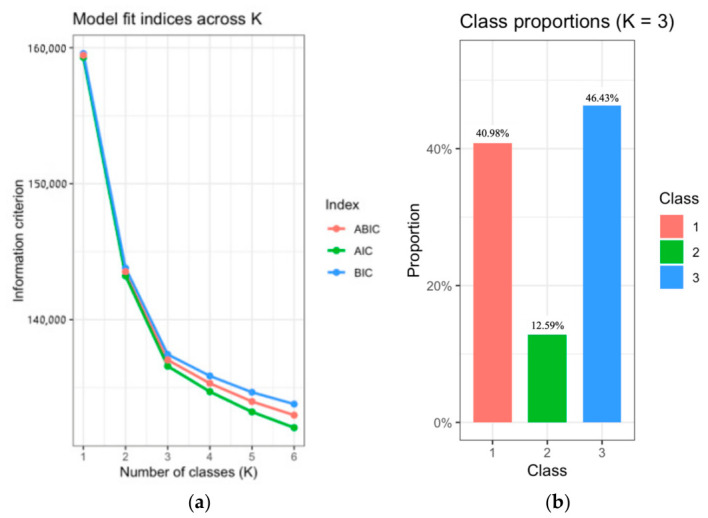
(**a**) Line Chart of Fit Metrics for Latent Class Analysis; (**b**) Classification Results of Latent Class Analysis.

This study identified grandparenting styles for left-behind children using 14 items and fitted latent class models with 1 to 6 categories, as shown in Table 2. While the log-likelihood improved and AIC, BIC, and adjusted BIC decreased as categories increased, indicating better fit, model selection also considered category size, parsimony, theoretical interpretability, and analytical feasibility (Nylund et al., 2007). Although the fit indices continued to improve from one to six classes, the improvements showed diminishing marginal gains beyond the three-class solution. More importantly, the three-class solution yielded theoretically meaningful and interpretable classes corresponding to distinct caregiving profiles characterized by high support with moderate control, low support with high negativity, and moderate levels across dimensions. Existing methodological studies have pointed out that excessively small categories often suggest that the number of categories may have been over-extracted. Such categories sometimes merely reflect specific patterns within the sample rather than stable underlying subgroups (Sinha et al., 2021). Simultaneously, small or rare categories are also more prone to instability in category recovery and parameter estimation (Nylund-Gibson & Choi, 2018). Consequently, although the 4-class, 5-class, and 6-class models continue to improve on certain fitting metrics, their minimum class proportions are only 0.027, 0.026, and 0.025, respectively—well below the minimum threshold recommended for stable class recovery. The 3-class model has an entropy of 0.894 and a minimum class proportion of 0.128 (see Figure 2), offering both high classification clarity and a good balance in class size. Therefore, the three-class solution was retained as the most parsimonious and theoretically grounded model.

**Figure 3:**
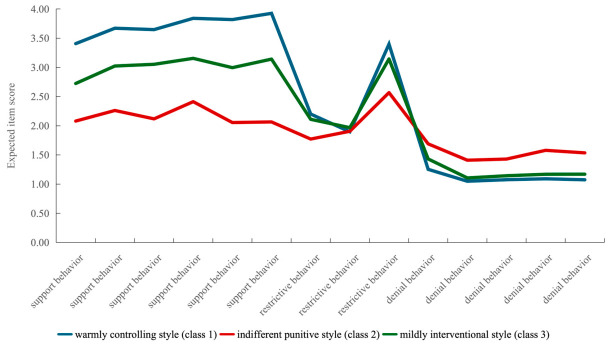
Mean item-response profiles across the three latent classes. Note: The items displayed on the x-axis correspond to the 14 grandparenting behavior indicators listed in Appendix Table A1.

According to latent class analysis, the three types of grandparenting styles exhibited distinct differences in the combination of supportive, controlling, and negative behaviors (see Figure 3). Class 1, the warmly controlling style, scored highest on supportive behaviors, including emotional comfort, parenting support, communication, and material provision, with moderate controlling behaviors and low negative behaviors. This combination of high support and moderate control is consistent with intensive parenting, a child-centered caregiving approach that is commonly observed in Chinese families (Craig et al., 2014; Lubiewska et al., 2025). Class 2, the indifferent punitive style, showed the lowest supportive behaviors and higher negative behaviors, such as physical discipline and rejection. Class 3, the mildly interventional style, exhibited moderate support, low negative behaviors, and mild control. The sample sizes were 2589 (40.98%), 795 (12.59%), and 2933 (46.43%) for Classes 1, 2, and 3, respectively.

After identifying latent categories, intergroup differences in psychological resilience and distress across grandparenting styles were further compared (shown in Table 3). Regarding psychological resilience, significant differences were found between categories (F = 677.069, *p* < 0.001). Children in the warmly controlling category exhibited the highest levels of psychological resilience; those in the indifferent punitive category the lowest, and those in the mildly interventional category intermediate levels. Post hoc tests confirmed all group differences were significant. Similarly, psychological distress differed significantly (F = 244.181, *p* < 0.001), with the warmly controlling style linked to the lowest distress, the indifferent punitive style linked to the highest, and the mildly interventional style linked to intermediate levels. All post hoc comparisons were significant. Overall, the warmly controlling style correlated with higher resilience and lower distress; the indifferent punitive style, the opposite, and the mildly interventional style intermediate. These findings support three statistically and substantively valid grandparenting style categories.

### 4.3. Grandparenting Styles, Parental Remote Parenting, and Children’s Mental Health

**Figure 4:**
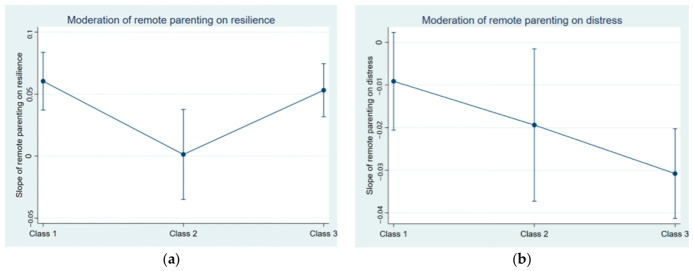
(**a**) Moderation of remote parenting on resilience; (**b**) Moderation of remote parenting on distress.

After identifying grandparenting style, we further explored their relationship with parental remote parenting and children’s mental health (see Figure 4). Regression models controlled for children’s gender, age, school stage, household registration, parents’ marital status, parents’ educational attainment, and county-level fixed effects. The mildly interventional style (Class 3), the largest group (46.43%), and the intermediate on parenting dimensions served as the reference. Model 1 included grandparenting styles; Model 2 further incorporated the control variables; Model 3 further included the frequency of parental remote parenting; and Model 4 included interaction terms to examine differences in the role of parental remote parenting across different grandparenting styles. The specific results are shown in Table 4.

Compared to the mildly interventional style, the warmly controlling style was significantly linked to higher psychological resilience, while the indifferent punitive style was significantly associated with lower resilience. This finding remained stable after controlling for covariates, supporting H1. When parental remote parenting was further included in Model 3, it showed a significant positive correlation with resilience (b = 0.047, *p* < 0.001), indicating that more frequent parental remote parenting corresponded to higher resilience of children. In Model 4, the interaction between warmly controlling style and parental remote parenting was not significant, suggesting that, compared with the mildly interventional style, there was no significant difference in the positive association. However, the interaction between the indifferent punitive style and remote parenting was negative and significant (b = −0.049, *p* < 0.05), indicating that the positive effect of remote parenting on resilience was weakened under this style. Simple slope analysis showed a significant positive association between parental remote parenting and resilience in the mildly interventional style (b = 0.053, *p* < 0.001, 95% CI [0.032, 0.074]) and warmly controlling style (b = 0.060, *p* < 0.001, 95% CI [0.037, 0.084]), but not in the indifferent punitive style (b = 0.001, *p* = 0.943, 95% CI [−0.035, 0.038]). The effect size for this interaction was small (Cohen’s f^2^ = 0.0013), explaining minimal additional variance. Thus, in grandparenting contexts marked by low support and negativity, the positive link between remote parenting and resilience disappears, partially supporting H2. Additionally, psychological resilience increased significantly with grade, as children in primary and secondary school showed higher resilience than preschoolers.

Compared to the mildly interventional style, the warmly controlling style was significantly associated with lower psychological distress, while the indifferent punitive style was significantly associated with higher psychological distress. These relationships remained stable after controlling for covariates, supporting H1. Model 7 shows that parental remote parenting was significantly negatively associated with psychological distress (b = −0.021, *p* < 0.001), indicating that more frequent remote parenting corresponded to lower distress. In Model 8, the interaction between the warmly controlling style and parental remote parenting was positive and significant (b = 0.022, *p* < 0.01), suggesting that the negative association between parental remote parenting and distress was weakened in the warmly controlling style. The interaction between the indifferent punitive style and parental remote parenting was not significant, indicating no difference from the mildly interventional style. Simple slope analysis revealed a significant negative association between parental remote parenting and distress in the mildly interventional style (b = −0.031, *p* < 0.001, 95% CI [−0.041, −0.020]) and indifferent punitive style (b = −0.019, *p* = 0.034, 95% CI [−0.037, −0.002]), but a weaker, non-significant association in the warmly controlling style (b = −0.009, *p* = 0.119, 95% CI [−0.021, 0.002]). The effect size for this interaction was small (Cohen’s f^2^ = 0.0022), indicating this weakening is modest. Thus, H2 was partially supported. Additionally, parental separation was consistently associated with higher psychological distress, suggesting that family structural instability may still contribute to children’s negative psychological experiences.

Regression analysis reveals a consistent link between grandparenting styles and children’s mental health. Parental remote parenting was also associated with higher psychological resilience and lower distress, but this varies by grandparenting style. The positive effect on resilience is weakened in the indifferent punitive style, while the negative effect on distress is reduced in the warmly controlling style. These findings highlight the need to consider the grandparenting context when evaluating parental remote parenting.

### 4.4. Sensitivity and Heterogeneity Analyses 

To eliminate confounding from young children’s comprehension limits or proxy-response bias, we re-estimated all regression models excluding preschoolers aged 3–6 years (*N* = 541). Table 5 shows that the significance, direction, and magnitude of all key interaction terms remained consistent with the main analysis, confirming the robustness of our primary findings.

To assess whether effects differed by developmental stage, we divided the sample into primary school (grades 1–6), junior high, and senior high (including vocational/technical schools) groups and re-estimated interaction models (shown in Table 6). Among primary school students, the interaction between the warmly controlling style and parental remote parenting was significant for psychological distress (b = 0.026, *p* < 0.01), but not for psychological resilience. In contrast, the interaction between the indifferent punitive style and parental remote parenting was significantly associated with psychological resilience (b = −0.059, *p* < 0.05), but not with psychological distress. Among junior high school students, only the interaction between the warmly controlling style and parental remote parenting was significant for psychological distress (b = 0.029, *p* < 0.05); none of the interaction terms for psychological resilience reached statistical significance. Among senior high, vocational, and technical school students, none of the interaction terms was statistically significant for either psychological resilience or psychological distress. Overall, these findings indicate that the moderating role of parental remote parenting was more evident among primary and junior high school students, whereas no clear moderation pattern emerged among older students.

## 5. Discussion and Conclusions

The question this study sought to explore was not merely whether grandparents were present, but how they were present, and how this presence may shape the role and meaning of parental remote parenting. The findings suggest that the grandparenting environments in which left-behind children are embedded are not homogeneous. The warmly controlling style is associated with higher psychological resilience and lower psychological distress, while the indifferent punitive style shows the opposite, and the mildly interventional style falls in between. Different configurations of supportive, controlling, and punitive behaviors thus situate children in everyday interactive contexts that differ in their relational quality. This extends recent discussions on the de-homogenization of grandparenting and highlights the importance of differentiating grandparenting contexts when exploring children’s psychological adaptation in intergenerational caregiving.

The warmly controlling style reflects a common parenting approach in China, especially within the rural–urban migration context, where grandparents often serve as primary caregivers due to parental labor migration. This pattern arises from structural factors like the hukou system and regional economic disparities, as well as strong cultural norms of intergenerational family responsibility. Consequently, grandparental care combined with parental remote parenting has become a typical family adaptation. Intensive parenting in these intergenerational arrangements emphasizes child-centered, high-involvement care characterized by constant attention, detailed planning, and active intervention beyond mere emotional support (Craig et al., 2014; Lubiewska et al., 2025). In Chinese families, this high-investment approach extends to grandparents through intergenerational coparenting (Luo, 2025). Thus, grandparenting styles should be seen as embedded roles within a shared caregiving system, often involving educational, supervisory, and disciplinary functions traditionally linked to parents (Harman et al., 2022; Kerrane et al., 2024; S. Wang, 2026).

From this intergenerational coparenting perspective, the warmly controlling style can be understood as high-involvement caregiving embedded within a warm relational context. Its key distinction lies in whether control is accompanied by responsive support and whether it is experienced by children as care and emotional recognition. Although both warmly controlling and mildly interventional styles show low negativity, they differ in relational engagement during daily interactions. Variations in children’s psychological adaptation across these styles likely reflect differences in intergenerational coordination quality rather than control alone. This implies that reducing negativity is insufficient for a supportive developmental environment. Children also need consistent, visible, and emotionally responsive engagement within the caregiving system (Britto et al., 2017; Pinquart, 2017).

From the perspective of children’s mental health, grandparenting styles show greater variation in relation to psychological resilience than psychological distress. This aligns with stronger associations between educational stage and resilience, which develops gradually through stable relationships, routines, and supportive interactions, accumulating over time (Masten et al., 2021). As primary caregivers of left-behind children, grandparents provide the most immediate relational context where caregiving styles may influence children’s emotional regulation, problem-solving, and sense of security (He et al., 2025). Thus, psychological resilience varies more across grandparenting styles. In contrast, psychological distress is more closely associated with concurrent negative emotional experiences and symptom burden (Ensel & Lin, 1991) and is sensitive to situational stressors like academic pressure and peer interactions, explaining its weaker association with grandparenting styles. These findings indicate that psychological resilience and distress are related but distinct aspects of children’s mental health. While increasing age may support the accumulation of coping resources, it does not necessarily imply a parallel reduction in distress. Therefore, attention to children’s mental health should encompass both the development of resilience and the mitigation of distress.

Intergenerational coparenting shows that parental remote parenting generally correlates with higher levels of children’s psychological resilience and lower distress, aligning with research on parent–child communication and digital connectivity. However, its effectiveness depends on the local caregiving context. In the indifferent punitive grandparenting style, the positive link to resilience weakens, while in the warmly controlling style, its association with reduced distress diminishes. Conversely, the mildly interventional style consistently supports children’s psychological adjustment through remote parenting. These differences likely stem from caregiving environments. Limited emotional support and negativity in the indifferent punitive style may hinder benefits, whereas ample care and engagement in the warmly controlling style lessen remote parenting’s impact. Thus, parental remote parenting should be viewed as a conditional relational resource embedded within grandparent-based caregiving. Building on research about parenting roles in migrant families (S. Wang, 2026), this study highlights the contexts where remote parenting serves as a protective factor.

This study, focusing on children aged 3–18, moves beyond traditional identity-based classifications of left-behind children (Dunifon, 2013; C. Zhou et al., 2021; Q. Zhou et al., 2026), which categorizes families by parental absence or grandparental care. Such categories inadequately explain why children under similar grandparenting experience different psychological outcomes. Addressing this, this research emphasizes intergenerational coparenting processes and the impact of digital communication on grandparenting (Jedovnicki, 2019). By viewing parent–child and grandparent–child relationships as nested, interdependent systems, it argues that children’s psychological adaptation depends on relational dynamics rather than fixed family types. Findings reveal that family processes affect psychological resilience and distress asymmetrically. Family relationships could simultaneously support adaptation and contribute to emotional burden, which do not always align. This highlights the importance of distinguishing developmental resources from situational distress in child outcomes. These results are context-specific to rural China’s intergenerational caregiving and require further validation in other cultural settings.

Grade-stratified analyses further revealed developmental variation in the moderating role of parental remote parenting. Among primary school students, the interaction between the warmly controlling style and parental remote parenting was significant for psychological distress, whereas the interaction between the indifferent punitive style and parental remote parenting was significant for psychological resilience. Among junior high school students, only the interaction between the warmly controlling style and parental remote parenting was significant for psychological distress. No significant interaction effects were observed among senior high, vocational, and technical school students. These findings suggest that the moderating role of parental remote parenting was more evident among primary and junior high school students. However, given the relatively small sample of senior high, vocational, and technical school students, the absence of significant interactions in this group should be interpreted cautiously.

In practical terms, supporting families with left-behind children requires greater attention to the interactions and alignment among multiple caregiving systems, including grandparents, parents, and children. Rather than focusing on a single caregiver, interventions may be more effective when they consider how different caregiving arrangements collectively shape children’s daily relational environments. For families characterized by an indifferent punitive style, priority should be given to reducing punitive, blaming, and rejecting behaviors in grandparents’ daily caregiving, while strengthening emotional responsiveness and basic supportive engagement. Without such improvements, the potential benefits of parental remote parenting may be limited. For families characterized by a mildly interventional style, more frequent parental remote parenting may be associated with additional gains in children’s psychological adjustment. In contrast, for families characterized by a warmly controlling style, the frequency of parental remote parenting may be less critical. Instead, future research should explore whether the quality of communication, particularly interactions involving emotional resonance, developmental guidance, and problem-solving, plays a more salient role.

Admittedly, although the study attempts to explore the impact of grandparenting styles on children’s mental health, several limitations remain. Cross-sectional data struggle to capture the dynamic relationships among grandparenting styles, parental remote parenting, and children’s mental health, and therefore do not permit causal inference. Additionally, parental remote parenting was measured primarily by frequency, reflecting intensity rather than the full multidimensional nature of remote parenting, without distinguishing among communication content, medium, and interaction quality. Future research could adopt a longitudinal design to simultaneously examine the intergenerational coparenting relationship between grandparents and parents, differences between paternal and maternal grandparents, and the burden of grandparenting styles within the current analytical framework. Moreover, future studies should distinguish between fathers’ and mothers’ remote parenting roles, as their influences on children’s development may differ. Such efforts would contribute to a more comprehensive understanding of intergenerational coparenting and children’s mental health in left-behind families.

## Figures and Tables

**Table 1:** Descriptive analysis.

**Continuous Variables**	***N***	**Mean (SD)**	**Min**	**Max**
Psychological resilience	6317	4.64 (0.97)	1	6
Psychological distress	6317	1.24 (0.45)	1	4
Parental remote parenting	6317	4.85 (1.64)	1	7
Age	6317	10.38 (3.00)	3	18
**Categorical Variables**	***N***	**Frequency**	**Percent**	**Cum.**
Gender	6317			
Male		3185	50.42	50.42
Female		3132	49.58	100
Grade	6317			
Preschool		541	8.56	8.56
Lower primary (grades 1–3)		1580	25.01	33.58
Upper primary (grades 4–6)		2390	37.83	71.41
Junior high school		1572	24.89	96.30
Senior secondary/Vocational secondary/Technical school		234	3.70	100
Household registration	6317			
Agricultural household registration		5999	94.97	94.97
Non-agricultural household registration		318	5.03	100
Parents’ marital status	6317			
Married		4755	75.27	75.27
Divorced		1408	22.29	97.56
Separated		154	2.44	100
Father’s level of education	6317			
Elementary school and below		389	6.16	6.16
Middle School		4419	69.95	76.11
High School		1260	19.95	96.06
Junior college		210	3.32	99.38
Undergraduate		36	0.57	99.95
Graduate and above		3	0.05	100
Mother’s level of education	6317			
Elementary school and below		445	7.04	7.04
Middle School		4552	72.06	79.10
High School		1075	17.02	96.12
Junior college		198	3.13	99.26
Undergraduate		38	0.60	99.86
Graduate and above		9	0.14	100

Note: The total of the percentages may vary slightly due to rounding.

**Table 2:** Model fit indices for the latent class analysis solutions (*N* = 6317).

k	logLik	npar	AIC	BIC	ABIC	Entropy	Min Prop	Max Prop
1	−79,604.5	42	159,293	159,576.5	159,443.1	—	1	1
2	−71,526.49	85	143,223	143,796.8	143,526.7	0.878	0.493	0.507
3	−68,162.95	128	136,581.9	137,446	137,039.3	0.894	0.128	0.463
4	−67,182.08	171	134,706.2	135,860.6	135,317.2	0.909	0.027	0.451
5	−66,393.69	214	133,215.4	134,660.1	133,980.1	0.889	0.026	0.402
6	−65,770.73	257	132,055.5	133,790.5	132,973.8	0.842	0.025	0.310

**Table 3:** Analysis of between-group differences in latent categories of grandparenting styles.

Mental Health	Class 1 M (SD)	Class 2 M (SD)	Class 3 M (SD)	F	Post-Hoc Comparison
Psychological resilience	5.074 (0.899)	3.884 (0.982)	4.456 (0.825)	677.069 ***	Class 1 > Class 3 > Class 2 (*p* < 0.001)
Psychological distress	1.135 (0.363)	1.519 (0.600)	1.245 (0.430)	244.181 ***	Class 2 > Class 3 > Class 1 (*p* < 0.001)

Note: Class 1 represents the warmly controlling style; Class 2 represents the indifferent punitive style; and Class 3 represents the mildly interventional style. *** *p* < 0.001. The post hoc comparison differences for psychological resilience were Class 2 − Class 1 = −1.190, Class 3 − Class 1 = −0.618, and Class 3 − Class 2 = 0.572; The corresponding differences for psychological distress were 0.384, 0.111, and −0.273, all with *p* < 0.001.

**Table 4:** Regression results.

	Model 1	Model 2	Model 3	Model 4	Model 5	Model 6	Model 7	Model 8
	Psychological Resilience	Psychological Resilience	Psychological Resilience	Psychological Resilience	Psychological Distress	Psychological Distress	Psychological Distress	Psychological Distress
Grandparenting styles (ref = Mildly interventional)								
Warmly controlling	0.618 ***	0.612 ***	0.593 ***	0.588 ***	−0.111 ***	−0.110 ***	−0.102 ***	−0.104 ***
	(0.023)	(0.024)	(0.024)	(0.024)	(0.011)	(0.011)	(0.011)	(0.011)
Indifferent punitive	−0.572 ***	−0.554 ***	−0.535 ***	−0.567 ***	0.273 ***	0.255 ***	0.246 ***	0.248 ***
	(0.038)	(0.038)	(0.039)	(0.042)	(0.023)	(0.023)	(0.023)	(0.025)
Parental remote parenting			0.047 ***	0.050 ***			−0.021 ***	−0.031 ***
			(0.008)	(0.010)			(0.004)	(0.006)
Warmly controlling × parental remote parenting				0.011				0.022 **
				(0.015)				(0.007)
Indifferent punitive × parental remote parenting				−0.049 *				0.011
				(0.023)				(0.013)
Gender (ref = Male)		−0.020	−0.017	−0.017		0.019	0.017	0.017
		(0.022)	(0.022)	(0.022)		(0.011)	(0.011)	(0.011)
Age		0.001	0.003	0.004		0.009 *	0.008	0.008
		(0.009)	(0.009)	(0.009)		(0.004)	(0.004)	(0.004)
Grade (ref = Preschool)								
Early elementary school (grades 1–3)		0.290 ***	0.285 ***	0.285 ***		−0.009	−0.006	−0.003
		(0.058)	(0.058)	(0.058)		(0.023)	(0.023)	(0.023)
Upper elementary grades (grades 4–6)		0.331 ***	0.326 ***	0.324 ***		−0.032	−0.030	−0.027
		(0.073)	(0.073)	(0.073)		(0.030)	(0.031)	(0.031)
Junior high school		0.345 ***	0.344 ***	0.342 ***		−0.063	−0.063	−0.061
		(0.095)	(0.095)	(0.095)		(0.040)	(0.040)	(0.040)
Senior secondary/Vocational secondary/Technical school		0.472 ***	0.467 ***	0.465 ***		−0.064	−0.062	−0.059
		(0.123)	(0.123)	(0.123)		(0.059)	(0.059)	(0.059)
Household registration (ref = Agricultural)		−0.074	−0.073	−0.068		0.038	0.037	0.037
		(0.052)	(0.051)	(0.052)		(0.029)	(0.029)	(0.029)
Parents’ marital status (ref = Married)								
Divorced		−0.092 ***	−0.005	−0.005		0.021	−0.018	−0.018
		(0.027)	(0.030)	(0.030)		(0.013)	(0.015)	(0.015)
Separated		−0.118	−0.025	−0.031		0.163 ***	0.122 **	0.123 **
		(0.064)	(0.065)	(0.066)		(0.044)	(0.045)	(0.045)
Father’s level of education (ref = Elementary school and below)								
Middle school		−0.068	−0.072	−0.073		−0.011	−0.009	−0.009
		(0.057)	(0.057)	(0.056)		(0.029)	(0.028)	(0.028)
High school		0.012	0.001	−0.000		−0.031	−0.026	−0.025
		(0.064)	(0.064)	(0.064)		(0.032)	(0.031)	(0.031)
Junior college		0.012	−0.004	−0.008		−0.083 *	−0.076	−0.078
		(0.098)	(0.098)	(0.098)		(0.042)	(0.042)	(0.042)
Undergraduate		−0.157	−0.162	−0.161		0.075	0.077	0.071
		(0.197)	(0.195)	(0.195)		(0.092)	(0.092)	(0.092)
Graduate and above		−1.080	−1.064	−1.041		0.423	0.416	0.401
		(0.721)	(0.754)	(0.738)		(0.692)	(0.674)	(0.668)
Mother’s level of education (ref = Elementary school and below)								
Middle school		0.050	0.039	0.041		−0.036	−0.031	−0.030
		(0.054)	(0.054)	(0.054)		(0.028)	(0.028)	(0.028)
High school		0.059	0.042	0.044		−0.009	−0.002	−0.001
		(0.065)	(0.065)	(0.065)		(0.033)	(0.033)	(0.033)
Junior college		0.023	0.003	0.006		−0.018	−0.009	−0.007
		(0.101)	(0.101)	(0.101)		(0.045)	(0.045)	(0.045)
Undergraduate		0.116	0.092	0.080		−0.025	−0.014	−0.018
		(0.148)	(0.149)	(0.148)		(0.086)	(0.087)	(0.086)
Graduate and above		−0.206	−0.244	−0.233		0.229	0.245	0.243
		(0.324)	(0.335)	(0.335)		(0.344)	(0.341)	(0.342)
County fixed effects	NO	YES	YES	YES	NO	YES	YES	YES
Constant	4.456 ***	4.439 ***	4.510 ***	4.509 ***	1.245 ***	1.008 ***	0.976 ***	0.956 ***
	(0.015)	(0.100)	(0.101)	(0.102)	(0.008)	(0.048)	(0.048)	(0.048)
*N*	6317	6317	6317	6317	6317	6317	6317	6317
R^2^	0.177	0.213	0.217	0.218	0.072	0.102	0.106	0.108

Note: Standard errors are in parentheses. * *p* < 0.05, ** *p* < 0.01, *** *p* < 0.001.

**Table 5:** Sensitivity analysis excluding preschool children.

	Full Sample (*N* = 6317)		Excluding Preschool Children (*N* = 5776)	
	Psychological Resilience	Psychological Distress	Psychological Resilience	Psychological Distress
Grandparenting styles (ref = Mildly interventional)				
Warmly controlling	0.588 ***	−0.104 ***	0.586 ***	−0.104 ***
	(0.024)	(0.011)	(0.025)	(0.012)
Indifferent punitive	−0.567 ***	0.248 ***	−0.555 ***	0.246 ***
	(0.042)	(0.025)	(0.043)	(0.026)
Parental remote parenting	0.050 ***	−0.031 ***	0.050 ***	−0.031 ***
	(0.010)	(0.006)	(0.011)	(0.006)
Warmly controlling × parental remote parenting	0.011	0.022 **	0.010	0.024 **
	(0.015)	(0.007)	(0.015)	(0.008)
Indifferent punitive × parental remote parenting	−0.049 *	0.011	−0.044 *	0.011
	(0.023)	(0.013)	(0.021)	(0.011)
Controls	YES	YES	YES	YES
County fixed effects	YES	YES	YES	YES
Constant	4.509 ***	0.956 ***	4.508 ***	0.958 ***
	(0.102)	(0.048)	(0.105)	(0.049)
*N*	6317	6317	5776	5776
R^2^	0.218	0.108	0.219	0.108

Notes: Standard errors are in parentheses. Controls include gender, age, grade, household registration, parents’ marital status, and parents’ education. * *p* < 0.05, ** *p* < 0.01, *** *p* < 0.001.

**Table 6:** Heterogeneity analysis by educational stage.

	Primary School		Junior High School		Senior High/Vocational/Technical	
	Psychological Resilience	Psychological Distress	Psychological Resilience	Psychological Distress	Psychological Resilience	Psychological Distress
Grandparenting styles (ref = Mildly interventional)						
Warmly controlling	0.562 ***	−0.104 ***	0.653 ***	−0.114 ***	0.745 ***	−0.052
	(0.030)	(0.014)	(0.046)	(0.019)	(0.108)	(0.062)
Indifferent punitive	−0.557 ***	0.271 ***	−0.605 ***	0.261 ***	−0.665 *	0.305
	(0.049)	(0.031)	(0.093)	(0.048)	(0.263)	(0.163)
Parental remote parenting	0.059 ***	−0.031 ***	0.039 *	−0.037 ***	0.054	−0.041
	(0.011)	(0.007)	(0.019)	(0.010)	(0.044)	(0.038)
Warmly controlling × parental remote parenting	0.015	0.026 **	−0.005	0.029*	0.027	−0.049
	(0.019)	(0.009)	(0.028)	(0.012)	(0.071)	(0.058)
Indifferent punitive × parental remote parenting	−0.059 *	0.004	−0.032	0.029	−0.060	0.073
	(0.028)	(0.018)	(0.048)	(0.024)	(0.096)	(0.064)
Controls	YES	YES	YES	YES	YES	YES
County fixed effects	YES	YES	YES	YES	YES	YES
Constant	4.479 ***	1.244 ***	4.492 ***	1.236 ***	4.582 ***	1.227 ***
	(0.018)	(0.010)	(0.032)	(0.015)	(0.076)	(0.042)
*N*	3970	3970	1572	1572	234	234
R^2^	0.183	0.080	0.214	0.092	0.269	0.068

Notes: The heterogeneity analysis excludes preschool children; subgroup sample sizes sum to *N* = 5776. Standard errors are in parentheses. Controls include gender, age, household registration, parents’ marital status, and parents’ education. * *p* < 0.05, ** *p* < 0.01, *** *p* < 0.001.

## Data Availability

The data are not publicly available due to privacy and ethical restrictions involving minors and children in difficult circumstances. De-identified data may be made available from the corresponding author upon reasonable request and subject to approval by the relevant ethics committee and administrative authorities.

## Appendix A

**Table A1:** Items Assessing Grandparenting Behaviors (4-point Likert scale).

They punish me even for the smallest of mistakes.
When I encounter something that upsets me, I can feel that they are doing their best to comfort me.
They’re always telling me what to wear or how to look.
When going out to play, you must get their permission.
At home, I can feel that my status is clearly lower than that of the other children.
They’ll cook the food I like.
They hit me at the drop of a hat and won’t listen to my explanations.
They’re happy to chat with me.
When I feel nervous or anxious, I can feel their support.
They’ll give me pocket money of their own accord.
They used to punish me for no reason.
They told me that whenever I go home, I must tell them what I’ve been doing at school.
I feel like they don’t want to be with me.
They regularly buy me new clothes and shoes.

Note: The items were adapted from the Chinese version of the Parental Rearing Style Questionnaire (Yue et al., 1993). In this study, the referent was the grandparent(s) serving as the child’s primary caregiver(s), including paternal or maternal grandparents. The 14 items were used as indicators in the latent class analysis.

**Table A2:** Psychological Resilience Questionnaire (6-point Likert scale).

I am willing to embrace difficulties and challenges.
I believe that once I hit rock bottom, I’ll bounce back quickly.
I will persevere with things that are worthwhile.
I always strive to do my best in everything.
No matter how great the difficulty, I will press on.
I feel that life is very meaningful.
No matter how hard it gets, I’ll keep going.
I feel the support of those around me.
I’m willing to try new things.
I always look on the bright side of things.
I am tenacious in pursuing my goals.
I believe that hard work pays off.
I’m happy to spend a little extra time as long as it helps us achieve our goal.
I can face difficulties with a positive attitude.
I will keep learning new things.
I stand by my convictions.
I will treat every failure as an opportunity to learn.

**Table A3:** Depression-Anxiety-Stress Scale (4-point Likert scale).

I find it difficult to calm myself down.
I feel parched.
I don’t seem to feel any joy or contentment.
I feel short of breath (e.g., wheezing or unable to catch my breath).
I find it very difficult to get started on my work.
Do I often overreact to things?
I feel a tremor (e.g., my hands are shaking).
I often feel completely exhausted.
I worry about situations that might cause me to panic or make a fool of myself.
I feel I have nothing to look forward to soon.
I feel anxious.
I find it very difficult to relax.
I feel depressed and down.
I can’t stand being interrupted when I’m working.
I feel like I’m about to break down.
I cannot muster any enthusiasm for anything.
I feel I don’t deserve to be human.
I find myself getting angry very easily.
I experience an irregular heartbeat even when I am not engaging in any obvious physical activity.
I feel afraid for no reason.
I feel that life is meaningless.

**Table A4:** Frequency of remote parenting (7-point Likert scale, 1 = every day, …, 7 = never).

How often does your dad get in touch with you?
How often does your mum get in touch with you?
